# Retracted: The Effect of Social Cognitive Interaction Training on Schizophrenia: A Systematic Review and Meta-Analysis of Comparison with Conventional Treatment

**DOI:** 10.1155/2023/9837847

**Published:** 2023-01-18

**Authors:** BioMed Research International

*BioMed Research International* has retracted the article titled “The Effect of Social Cognitive Interaction Training on Schizophrenia: A Systematic Review and Meta-Analysis of Comparison with Conventional Treatment” [1] due to concerns that the peer review process has been compromised.

Following an investigation conducted by the Hindawi Research Integrity team [2], significant concerns were identified with the peer reviewers assigned to this article; the investigation has concluded that the peer review process was compromised. We therefore can no longer trust the peer review process and the article is being retracted with the agreement of the editorial board.
